# Exploring the genetic and phenotypic diversity within and between onion (*Allium cepa* L.) ecotypes in Morocco

**DOI:** 10.1186/s43141-022-00381-w

**Published:** 2022-07-03

**Authors:** Amal Brahimi, Sofie Landschoot, Boris Bekaert, Lhoussain Hajji, Hassan Hajjaj, Kris Audenaert, Geert Haesaert, Hamid Mazouz

**Affiliations:** 1 Biotechnologies and Valorization of Biological Resources Laboratory, Faculty of Science of Meknes, University of Moulay Ismail, P.B 11201 Zitoune, Meknes, Morocco; 2 Department of Plants and Crops, Faculty of Bioscience Engineering, Ghent University, Valentin Vaerwyckweg 1, 9000 Ghent, Belgium

**Keywords:** *Allium cepa* L., Genetic diversity, Genomics, ISSR, Phenotype

## Abstract

**Background:**

Gaining insight into crop diversity, both at the genetic and phenotypic levels, is of prime importance for onion breeding with an enhanced yield and quality in combination with improved resistance to biotic and abiotic stresses. In the current study, 192 different onion plants, representing 16 ecotypes, were characterized using ISSR markers.

**Results:**

Based on the ISSR marker profile, there was a clear grouping of the plants into 16 different ecotypes. Though the 16 populations originated from the same geographic region in Morocco, a significant genetic diversity was detected. After a genomic characterization, field trials in three different environments in Morocco were laid out. The phenotypic characterization showed that there were always significant differences between ecotypes, and for most traits, there was also a significant environmental effect and a significant interaction between environment and ecotype. The broad-sense heritability (*H*^2^) for the phenotypic traits associated with color (L*, a*, and b*) was the largest (84.2%, 80.6%, 79.2%), demonstrating that color is conditioned primarily by genetic factors. In contrast, the *H*^2^ for yield was the lowest (41.8%), indicating that the environment has a substantial effect on yield. In addition, there was a significant association between the presence/absence of certain bands and various phenotypic traits.

**Conclusion:**

ISSR markers are a powerful tool in distinguishing onion ecotypes. In addition, significant associations between marker scores and phenotypic traits could be detected, representing particular importance for future breeding programs.

## Background

With a size of 31,862 ha and an annual production of 828,901 tons, onion (*Allium cepa* L.) is one of the main vegetable crops grown in many regions of Morocco [1]. Each region has its specific onion ecotype, which is a genetically distinct population that has genetic characteristics adapted to its geographic origin.

Besides its global culinary significance, onion has plenty of medicinal benefits thanks to the presence of flavonoids and alk(en)yl cysteine sulphoxides. These compounds have been reported to having powerful positive health effects, including anti-carcinogenic, anti-thrombotic, anti-asthmatic, and antibiotic effects [2]. Despite its global economic importance, genetic research on onion lags behind that of other vegetable crops [3]. This can mainly be attributed to its biennial life cycle, high inbreeding depression, outbreeding, high level of heterozygosity, cross-pollinating nature, large genome size (16.3 Gbp), and rarity of selective markers [4, 5]. Nevertheless, in the current study which outlines the de novo assembly, using the doubled haploid onion line “DHCU066619,” a high-quality onion reference genome (14.9 Gbp) was created. It consists of eight pseudomolecules, with five genetic linkage maps [4].

According to Walters et al. [6], crop domestication and breeding practices have resulted in a significantly reduced genetic diversity of many vegetable crops around the world. In Morocco (Sous Massa, a mountainous region of southwestern), despite the fact that a significant loss of vegetable crop landraces was detected in the last 30 years, many other vegetable crop landraces are still largely farmed, including onion [6]. The presence and the preservation of these crop landraces provide an important source of valuable traits needed for climate-resilient breeding [6], which is particularly important for semiarid regions that will encounter several environmental stresses in the future [7].

As gaining insight into the genetic variability is of pivotal importance, modern breeding employs marker analysis to explore populations to tackle the loss of valuable genetic traits [8]. The main objective of plant breeding is the improvement of crop yield and productivity over a long period. It started with phenotypic markers related to the visual traits, but these traits are not always transmitted to the next generation. In the next stage, biochemical markers (e.g., protein profile) were used for selecting the plant of interest. However, it has been proven that phenotypic markers are not efficient since the outcome is influenced by the environment [9–11].

To deal with this influence, genetic markers present a lot of advantages over phenotypic markers for two main reasons. Firstly, they are not influenced by the environment [12, 13]. Secondly, they are highly polymorphic and stable across the development cycle [14].

Several molecular techniques serve to characterize the genetic diversity of crop plants. The most used methods are random amplified polymorphic DNA (RAPD), restriction fragment length polymorphism (RFLP), single nucleotide polymorphism (SNP), amplified fragment length polymorphism (AFLP), inter-simple sequence repeat (ISSR), and microsatellite or simple sequence repeat (SSR) [11].

Each molecular marker presents some advantages: e.g., the low cost and the simplicity of RAPD and ISSR, the high genome coverage and the large number of bands for AFLP, and the revelation of variability at the allelic level using RFLP and SSR markers [11, 14]. Despite the benefits of these molecular techniques, they are limited by low reproducibility: e.g., for RAPD, the high cost of AFLP, SSR, and RFLP, in addition to the necessity of specific primers for SSR [15].

Inter-simple sequence repeats (ISSR) have been proven to be very useful for the analysis of genetic diversity in plant species. ISSR analysis is a fast, accessible technique with a high level of polymorphism and reproducibility due to the use of long primers (16–25 bp) [15]. The development of ISSR primers does not require prior knowledge of a specific segment, because the tandem repetition is common in all vegetal species [16].

ISSR markers are widely used in plant genomic analysis. It is used for the estimation of genetic diversity within many crops like *Eleusine coracana* [17], *Oryza* genus [18], and *Carthamus tinctorius* [19] and the identification of the relationship degree between cultivar and varieties, in addition to the linking of DNA markers with high valuable agronomic characteristic [20].

In Morocco, there are two main climatic zones: the Mediterranean northern coastal regions and the southern interior regions. This climatic separation results from the presence of the Atlas Mountain range in the center of Morocco. In the northern regions, the rainy season runs from November to March, with an average of 50 to 100 mm of rainfall per month. The temperature varies between 22 and 25 °C in the summer and between 10 and 12 °C in the winter. In the Atlas Mountains, especially at higher altitudes, the temperatures in the wet season are lower than in the coastal regions. However, in the southern and interior regions, the temperatures vary from 25 to 30 °C in the summer and drop to less than 15 °C in the winter [21].

Given the importance of onion and the lack of knowledge of its genetic diversity in Morocco, the main goals of the present study include (i) characterization for the first time of the genetic diversity within and between 16 Moroccan *A. cepa* L. ecotypes using ISSR markers, (ii) study the efficiency of ISSR markers to gain insight into Moroccan onion ecotypes structure, and (iii) gaining insight into the phenotypic diversity expressed under different growing conditions.

## Methods

### Plant material collection

In collaboration with the National Office of Agricultural Advices (ONCA) and the Agricultural Development Regional Office (ORMVA), 18 Moroccan onion ecotypes (*A. cepa* L.) were collected as seeds from 14 different regions around the country (Table 1).

**Table 1:** Geographic origin of collected onion ecotype seeds (National Meteorology Directorate of Morocco, 2018)

Ecotypes	Region	Origin	Altitude (m)	Annual temperature (°C)	Annual rainfall (mm)
**P1**	Béni Mellal-Khénifra	Souk Sabt	421	11–32	38.5
**P2**	Béni Mellal-Khénifra	Zaouit Echikh	1442	13–32	40.5
**P3**	Béni Mellal-Khénifra	Souk Sabt	421	11–32	38.5
**P4**	Tanger-Tétouan-Al Hoceïma	Tetouane	192	13–27	30.8
**P5**	Marrakech-Safi	Youssoufia	269	15–31	21.4
**P6**	Casablanca-Settat	Doukkala	61	15–26	32.0
**P7**	Casablanca-Settat	El gara	305	13–31	30.4
**P8**	Casablanca-Settat	Berrechid	230	14–30	29.7
**P9**	Fes- Meknés	Agourai	894	12–32	58.8
**P10**	Fes- Meknés	Agourai	894	12–32	58.8
**P11**	Fes- Meknés	Hajeb	1190	7–24.5	57.2
**P12**	Fes- Meknés	Bouderbala	768	7–24.5	57.2
**P13**	Fes- Meknés	Ain karma	273	12–33	58.8
**P14**	Fes- Meknés	Guigou	1524	4.7–23.2	37.2
**P15**	Fes- Meknés	Guigou	1524	4.7–23.2	37.2
**P16**	Draa-Tafilalet	Goulmima	1018	10–35	20.7
**P17**	Béni Mellal-Khénifra	Ait Malek	1195	13–33	21.9
**P18**	Fes- Meknés	Bouderbala	768	7–24.5	57.2

The geographic localization of onion ecotypes varied between 31.401200 N; 8.63339 W and 34.003204 N; 4.88375 W and between 61 and 1524 m above sea level.

It is important to state that seeds were only collected from farmers who produced them by themselves. To preserve the viability of the seeds, each population’s seeds have been put in labeled bags made of non-transparent fabric, ensuring good aeration and preventing direct contact with light. All the bags were stored under the same conditions.

### Germination test

Superficial sterilization was made by immersing the seeds in a solution of sodium hypochlorite (6.25 g/L) for 10 min, stirring, and then rinsing them twice with sterile demineralized water for 5 min. To evaluate the seed quality and viability, a germination test was carried out according to the International Rules for Seed Testing (ISTA) [22]. With three replicates, 100 seeds were placed in Petri dishes containing blotting paper moistened with sterile water and then incubated at 25 °C. Assessments of germinated seeds started after 2 days and continued until the tenth day. For the reliability of the germination test, it is necessary to not select the chosen seeds and to not discard small, malformed seeds since the sample must represent the entire seed lot.

### Molecular screening

#### DNA extraction

From each ecotype, 12- to 20-day-old seedlings were harvested, and the plant material (~ 30 mg/plant) was ground in liquid nitrogen using a pestle and a mortar. The genomic DNA extraction from the ground onion plant was done using the Invisorb® Spin Plant Mini Kit (Stratec Molecular). DNA concentrations were measured using the Quantus™ Fluorometer (Promega). The optical density was determined at 260 and 280 nm against TE buffer as blank, and the ratio of A260 to A280 was calculated to check the purity of the DNA. It is important to note that the DNA samples for the analysis were diluted to 50 ng/μL with the molecular-grade water.

#### ISSR amplification

The isolated genomic DNA was amplified with ten University of British Columbia (UBC) ISSR primers (Table 2). The amplifications were performed individually and repeated twice. The total reaction volume of the master mix was 25 μL, and it was composed of 1 μL template DNA (50 ng/μL), 5 μL GoTaq® Green Reaction Buffer (5 ×), 1 μL nucleotide mix (10 mM each), 0.25 μL of GoTaq® DNA Polymerase (5 U/μL) (Promega, Belgium), 2 μL primer (0.05 mM) from Integrated DNA Technologies (IDTDNA, USA), and 15.75 μL molecular-grade water. The amplification of genomic DNA with the master mix was performed in a PCR machine (Gene Amp® PCR System 97). The initial denaturation was at 94 °C for 4 min, and the amplification was carried out with 40 cycles, which started with an initial denaturation at 94 °C for 30 s and the annealing temperature varied for each primer (Table 2). Each cycle finished with an extension at 72 °C for 60 s, and the PCR reaction ended with a final extension at 72 °C for 7 min. The amplified products were separated by gel electrophoreses in agarose gel (1.5%) in 1 × TBE buffer under 80 V for 105 min, and then the gels were stained with EthBr and visualized by Molecular Imager® Gel Doc XR System.

**Table 2:** Primer name, primer sequence, and annealing temperature (°C)

Primer	Sequence repeat	Annealing T (°C)
UBC811	GAGAGAGAGAGAGAGAC	45.80
UBC815	CTCTCTCTCTCTCTCTG	45.80
UBC823	TCTCTCTCTCTCTCTCC	40.90
UBC825	ACACACACACACACACT	42.90
UBC826	ACACACACACACACACC	42.90
UBC835	AGAGAGAGAGAGAGAGCC	40.00
UBC840	GAGAGAGAGAGAGAGATT	40.00
UBC841	GAGAGAGAGAGAGAGATC	42.90
UBC842	GAGAGAGAGAGAGAGATG	45.80
UBC846	CACACACACACACACAGT	40.00

### Phenotypic measurements

Next to genetic screening, the differences between phenotypes of the various ecotypes were determined. Therefore, the different ecotypes were grown in three different regions: (i) Goulmima (31.69166667 N, 4.95916667 W and 1018 m of altitude), an oasis region in the desert environment; (ii) Ifrane (33.533333 N, 5.100000 W and 1664 m of altitude) in the middle of the Atlas Mountain environment; and (iii) Ras Ijerri (34.311944 N, 6.006389 W and 598 m of altitude) in the Saïs plateau. The planting started in the second week of November 2018 with a broadcast sowing of 16 onion ecotype seeds, in a randomized complete block design with three replicates, laid out in 48 experimental units of 1 m^2^. The quantity of seed per unit per ecotype was calculated using the germination rate and the weight of 1000 seeds to have 1000 onion bulbs in each unit. Four months later, only onion plantlets with a good vegetative appearance were transplanted into a new unit of 2 m^2^ in randomized complete blocks with three replicates. The agricultural practices (fertilization, irrigation, and maintenance) were the same for all the units in the same location and were according to the crop husbandry practices of Moroccan farmers.

After harvesting (August 2019), various characteristics of the onion were measured for randomly selected onion bulbs from each unit for all ecotypes in each location. These characteristics include the diameter in two directions using a caliper, the weight of the bulbs, the total yield, and the number of onions per kilogram. In addition, the bulb shape index (BSI) was calculated as the ratio of polar diameter to the equatorial diameter. Furthermore, the coloration of the outer layers (the skin) was perceived using the KONICA MINOLTA, Chroma meter CR-5. It is based on the L*a*b* colorimetric model (also known as the CIE Lab), in which a color is indicated by three values: L*, luminance, expressed as a percentage (0 for black to 100 for white); a* and b* two color ranges, from green to red and from blue to yellow, respectively, with values ranging from − 120 to 120.

### Statistical analysis

The R software package [23] was used to analyze the data and to make graphs. To create a binary matrix, amplified reproducible bands were scored as present (1) or absent (0) using the GelAnalyzer 2010 software.

To gain an insight into the informativeness and discriminative power of the ISSR markers, several parameters of the banding pattern were determined, for example, the total number of bands, percentage of polymorphic bands, and the polymorphic information content (*PIC*). The latter was calculated as ${ \mathrm{PIC}}_{i}=2 x {f}_{i}(1- {f}_{i})$, where PIC_*i*_ is the polymorphic information content of marker *i*′, *f*_*i*_ is the frequency of the amplified allele (band present), and *1 − f*_*i*_ is the frequency of the null allele [24]. To detect the genetic similarity between the genotypes, the binary matrix was converted to a Jaccard distance [25] matrix with the pairwise distances between the onion genotypes. Based on these distances, a neighbor joining algorithm was used to construct a dendrogram.

To verify whether or not a correlation existed between the genetic distance and the distance between the sampling sites of the plants, a Mantel test with 1000 permutations was used. This test checks for significant correlations between distance matrices by a permutation procedure. A linear discriminant analysis (LDA) was applied to determine whether, based on the banding pattern of the different onion genotypes, a differentiation up to the ecotype level was possible. Based on LDA, linear combinations of ISSR bands that provide the best separation between populations can be found.

To analyze the effects of ecotypes and the environment on phenotype expression, a two-way analysis of variance (ANOVA) was used. Based on the output of the ANOVA, the broad-sense heritability (*H*^2^) was calculated as *H*^2^ = *sg*^2^/(*sg*^2^ + *sge*^2^/*n* + *se*^2^/*nr*), in which *sg*^2^, *sge*^2^, *se*^2^, *r*, and *n* represent the estimated variances for the genetic effects, genotype-environment interaction effects, random errors, number of replicates, and number of environments, respectively.

A principal component analysis (PCA) was used to detect the grouping of the phenotypes, and a Spearman rank correlation was calculated to reveal the association between molecular markers and the phenotypic traits.

## Results

### Germination test

**Fig. 1:**
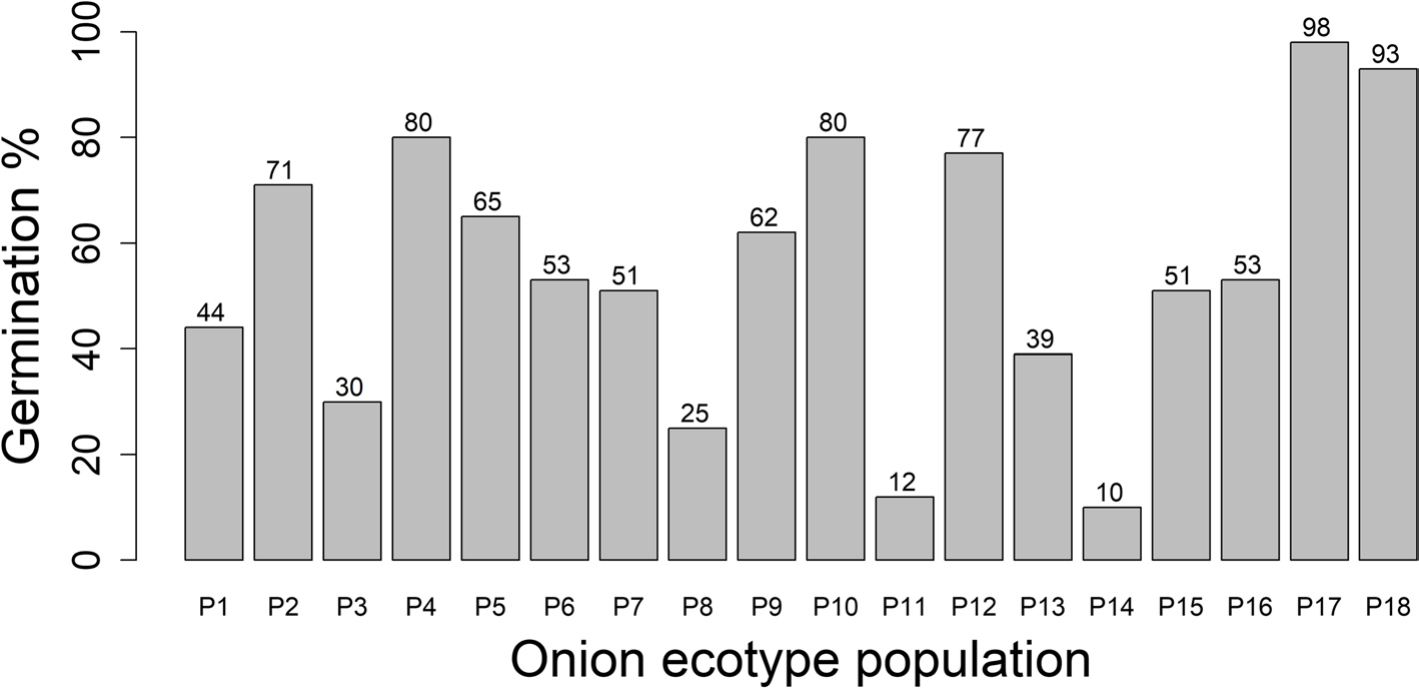
Germination rate results of 18 onion ecotypes from Morocco (R Core software)

The germination test revealed that there were clear differences in seed viability between ecotypes. Genotypes of ecotypes 11 and 14 resulted in an average germination percentage of 12% and 10%, respectively (Fig. 1). Due to this low germination percentage, these ecotypes were excluded from the further analysis.

### Genetic diversity

**Fig. 2:**
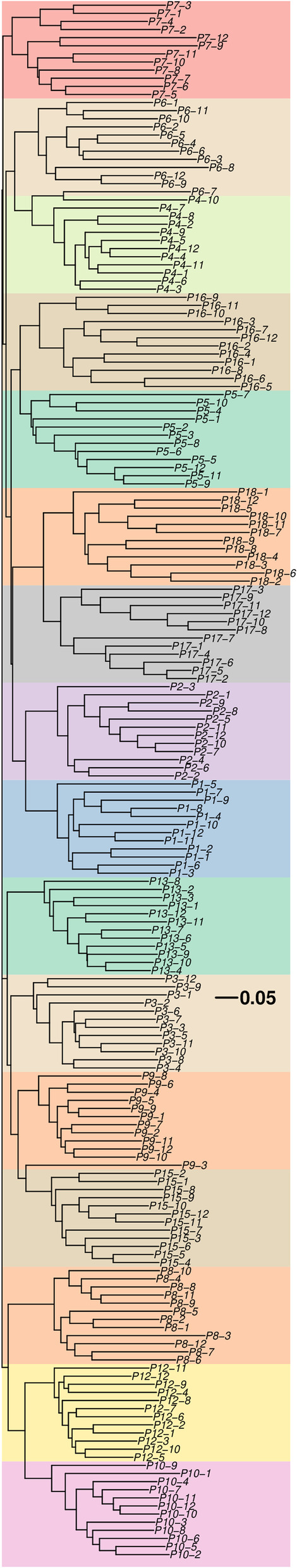
Neighbor joining tree of the 192 onion plants (R Core software)

The screening of the 192 onion plants with 10 ISSR markers revealed a clear subdivision into the 16 different populations as shown by the dendrogram in Fig. 2.

The statistical significance of this subdivision was proven by a *p*-value ≪ 0.01 of an analysis of molecular variance (AMOVA). According to the AMOVA, 58% of the total variance could be attributed to variations between populations, whereas 42% of the variance was within-population variance. A pairwise post hoc comparison revealed that all the populations significantly differed from each other based on their ISSR profile (*p*-values pairwise comparisons ≪ 0.01). Furthermore, in Fig. 2, it can be concluded that the diversity within the populations differs from one to another.

In Table 3, the total number (and %) of polymorphic fragments for each ecotype was given together with the average Jaccard distance.

**Table 3:** Number of fragments, percentage of polymorphic fragments, and average Jaccard distance for each ecotype

Ecotypes	No. of fragments	% polymorphic	Jaccard distance
P1	79	93.67	0.417
P2	58	91.38	0.488
P3	72	90.28	0.497
P4	56	83.93	0.427
P5	55	98.18	0.617
P6	62	95.16	0.560
P7	64	95.31	0.658
P8	82	89.02	0.498
P9	67	89.55	0.428
P10	66	74.24	0.362
P12	64	79.69	0.377
P13	44	88.64	0.437
P15	41	85.37	0.465
P16	28	100.00	0.722
P17	33	90.91	0.538
P18	22	100.00	0.745

The individuals belonging to populations 16 and 18 were most diverse since all bands are polymorphic, i.e., none of the bands was present in each individual of the population. In addition, these populations have the highest average within-population Jaccard distance, 0.72 and 0.75, respectively. In contrast, population 10 is characterized by the lowest frequency of polymorphic bands (74%) and the lowest within-population Jaccard distance (0.118).

**Fig. 3:**
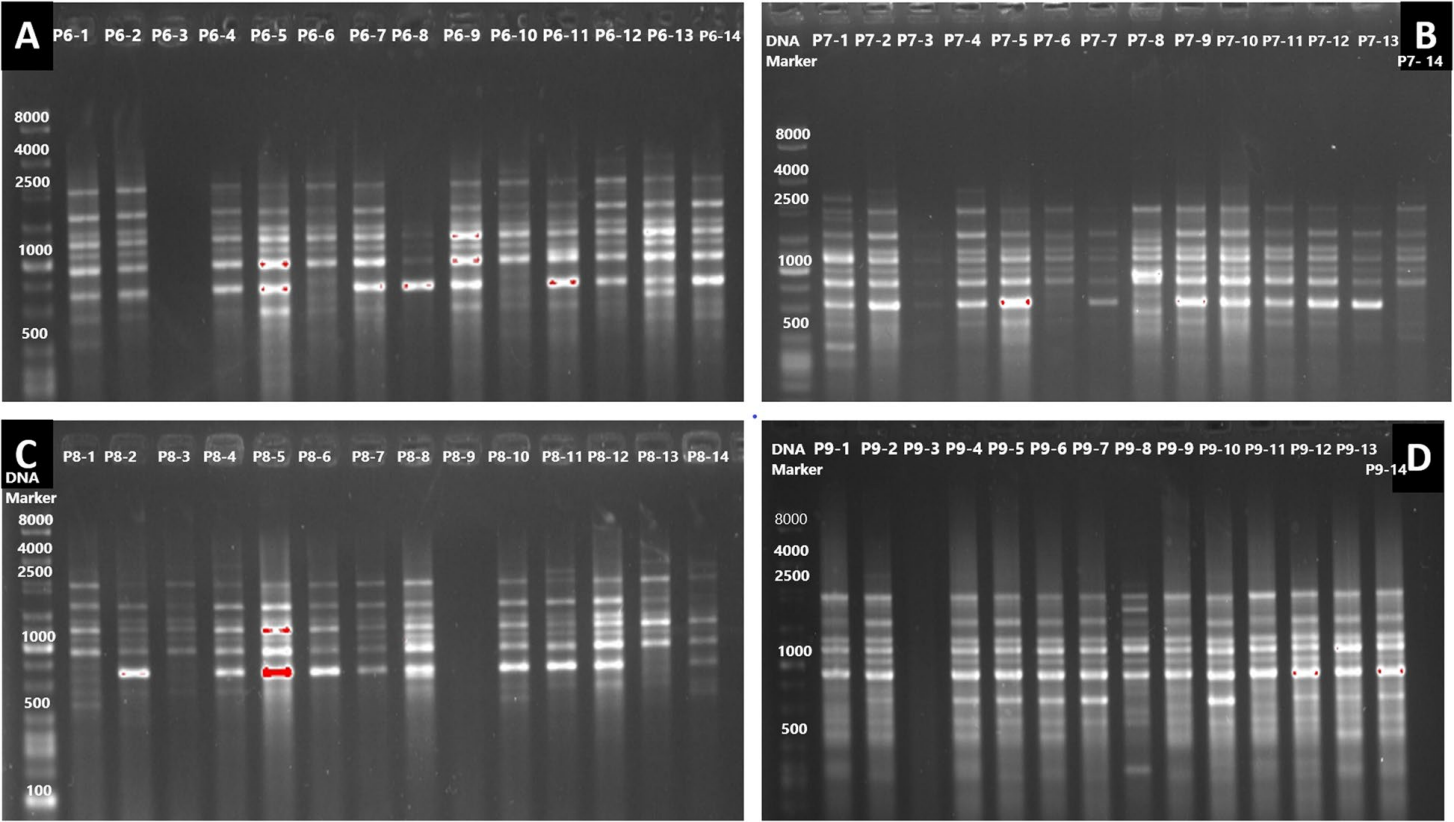
Allelic pattern of UBC 835 ISSR marker studies in 14 onion plants from different populations. **a** P6. **b** P7. **c** P8. **d** P9 (R Core software)

The informativeness and the discriminative power of the different markers are presented in Table 4. All fragments are polymorphic, i.e., none of the bands is present in each individual (Fig. 3). The UBC 826 marker had the highest PIC value (0.368) and therefore was the best marker for examining the genetic diversity between individuals.

**Table 4:** Marker parameters calculated for selected ISSR primers

Primer	No. of fragments	% polymorphic fragments	mean PIC	SD PIC
UBC811	17	100	0.296	0.135
UBC815	12	100	0.161	0.120
UBC823	10	100	0.195	0.110
UBC825	15	100	0.229	0.175
UBC826	8	100	0.368	0.166
UBC835	14	100	0.332	0.189
UBC840	18	100	0.323	0.189
UBC841	8	100	0.284	0.129
UBC842	15	100	0.179	0.130
UBC846	9	100	0.323	0.206

*SD* Standard deviation, *PIC* Polymorphic information content

**Fig. 4:**
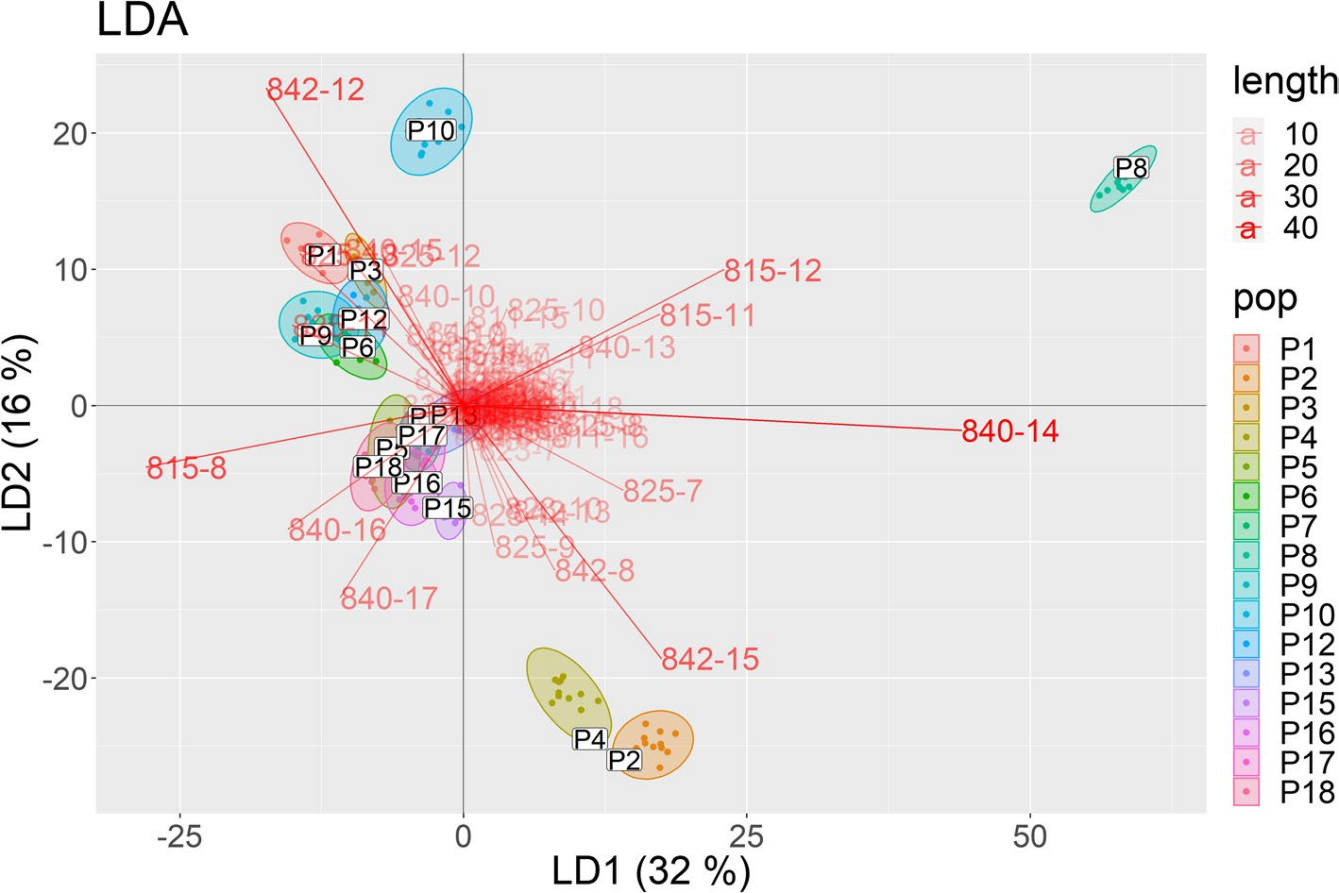
Genetic variation of the 192 onion plants visualized along the first two linear discriminant axes (LDA) (R Core software)

In the next step, an LDA was applied to identify the bands that were characteristic of a population. In Fig. 4, population P8 lays quite far from the others in the LDA biplot. However, none of the remaining populations had a band at position 14 for marker UBC 840, whereas one of the members from population P8 had a band at this position. Here, we conclude that this marker can be used to distinguish population P8 from the other populations; also, based on this analysis, populations P5, P7, P13, P17, P18, P16, and P15 form a cluster as well as P1, P3, P6, P9, and P12 form a cluster. Without cross-validation, a classification accuracy of 100% was obtained, indicating that each onion plant was assigned to the correct ecotype. If a leave-one-out cross-validation was applied, the accuracy dropped to 93%.

**Fig. 5:**
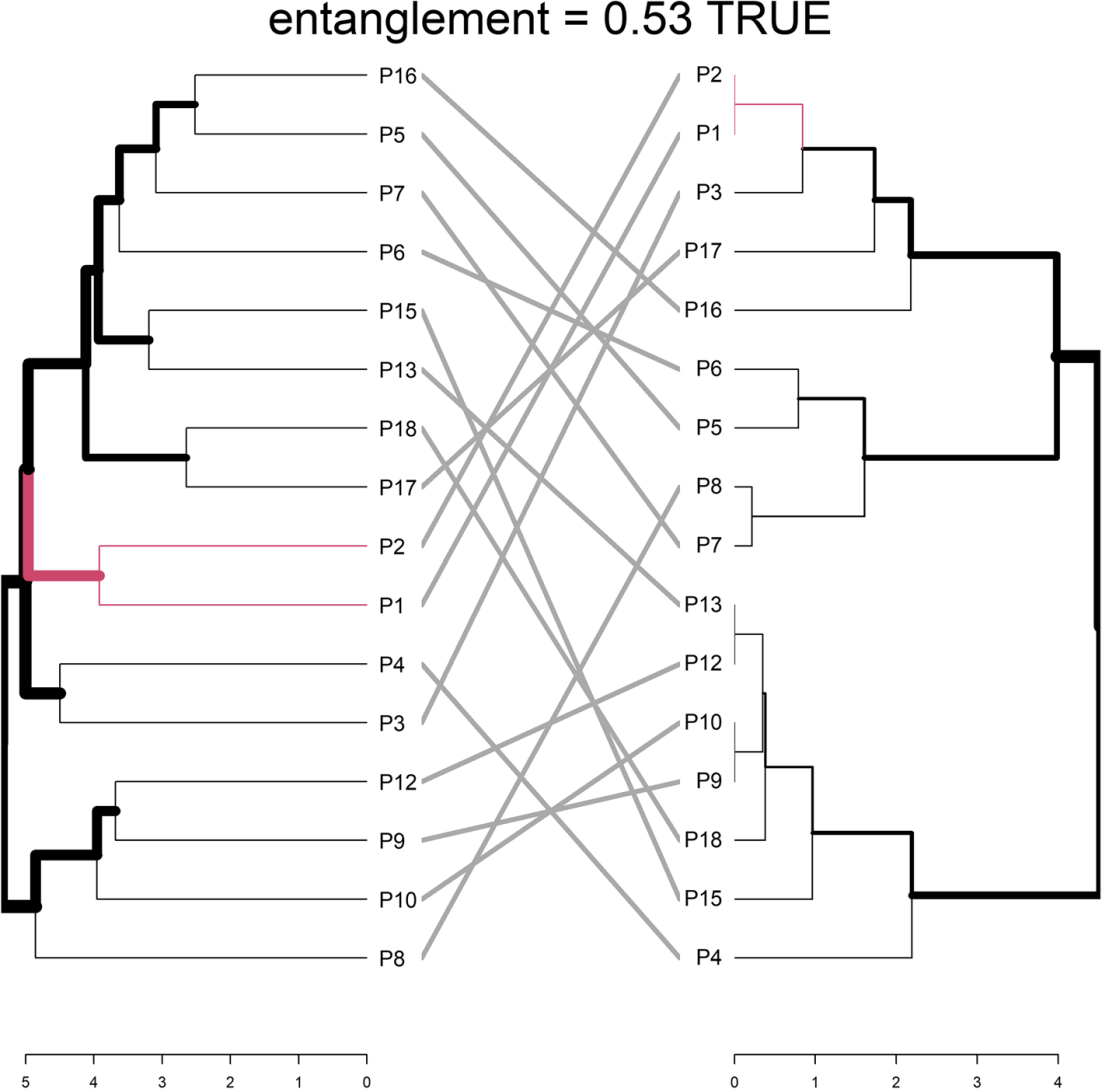
Comparison of the clustering of the populations based on genotype (left) and origin (right) (R Core software)

Based on the comparison between the geographic distance and the genetic distance of each individual, populations P1 and P2 are most similar (Fig. 5).

It appears also clear that populations P5, P6, and P7 cluster together based on the ISSR marker profile and based on the geographic distance. The similarity level between the genetic profile tree and the origin distance tree, based on the entanglement, which is the measurement of the similarity between two trees, is 0.53. However, based on a Mantel test, the correlation between the geographic distances and the genetic distances was not significant (*p*-value 0.147).

### Phenotype diversity

The average of the phenotypic traits is presented in Table 5. A two-way ANOVA was used to detect the effects of ecotype and the environment on the various phenotypes.

**Table 5:** Average different phenotypic traits of onion ecotypes by region

Region	IFRANE	RAS IJERRI	GOULMIMA
Pdia (mm)	40.5–86.4	46.2–89.3	33.5–61.0
Edia (mm)	54.5–87.6	61.4–97.0	33.6–72.5
Bulb weight (g)	58.2–264.5	81.7–394.1	29.1–108.3
*L* ^*^	43.7–81.7	45.3–81	36.5–87
*a* ^*^	(-0.6)–17.6	(-0.6)–17.2	0.2–16.4
*b* ^*^	9.5–26.0	10.0–24.0	5.0–30.1
Yield (t/ha)	–	16.0–60.0	4.4–23.4
Shape	0.9–1.37	1.01–1.5	0.64–1.6

*Pdia* Polar diameter, *Edia* Equatorial diameter, *BulbW* Weight of the onion bulb, L^*^, a^*^, b^*^ coloration compounds

The measurements revealed that for the coloration component a* and b* of the CIEL*a*b* color space, there seemed to be no significant effects of region and no significant interactions between regions and ecotypes only for the component a* (Table 6).

**Table 6:** Results of the ANOVA tests for the different phenotypic traits, with the *H*.^2^ values

	**Pdia**	**Edia**	**Shape**	**BulbW**	**Yield**	***n*/kg**	L^*^	a^*^	b^*^
Ecotype	< 0.01	< 0.01	< 0.01	< 0.01	< 0.01	< 0.01	< 0.01	< 0.01	< 0.01
Region	< 0.01	< 0.01	0.001	< 0.01	< 0.01	< 0.01	< 0.01	0.763	0.432
Ecotype × region	< 0.01	< 0.01	< 0.01	< 0.01	< 0.01	< 0.01	< 0.01	0.060	< 0.01
*H*^2^	48.6	55.0	69.8	60.4	41.8	45.1	84.2	80.6	79.2

*Pdia* Polar diameter, *Edia* Equatorial diameter, *BulbW* Weight of the onion bulb, *n/kg* Number of onions per kilogram, L^*^, a^*^, b^*^ coloration compounds

Thus, for the latter, the response of the ecotypes for two phenotypic traits is the same across environments, whereas for the other traits, the response of the ecotypes significantly differs between regions (environment effect), and the ecotypes react differently depending on the environment (interaction effect). In addition, the two-way ANOVA revealed that there were always significant differences between the ecotypes (*p*-values ≪ 0.01) (Table 6).

The broad-sense heritability (*H*^2^) for the phenotypic traits associated with color (L*, a*, and b*) was the largest (79–84%), demonstrating that onion color is primarily determined by genetic factors. In contrast, the *H*^2^ for yield (42%) was the lowest, indicating the environment has a substantial effect on the yield (Table 6).

**Fig. 6:**
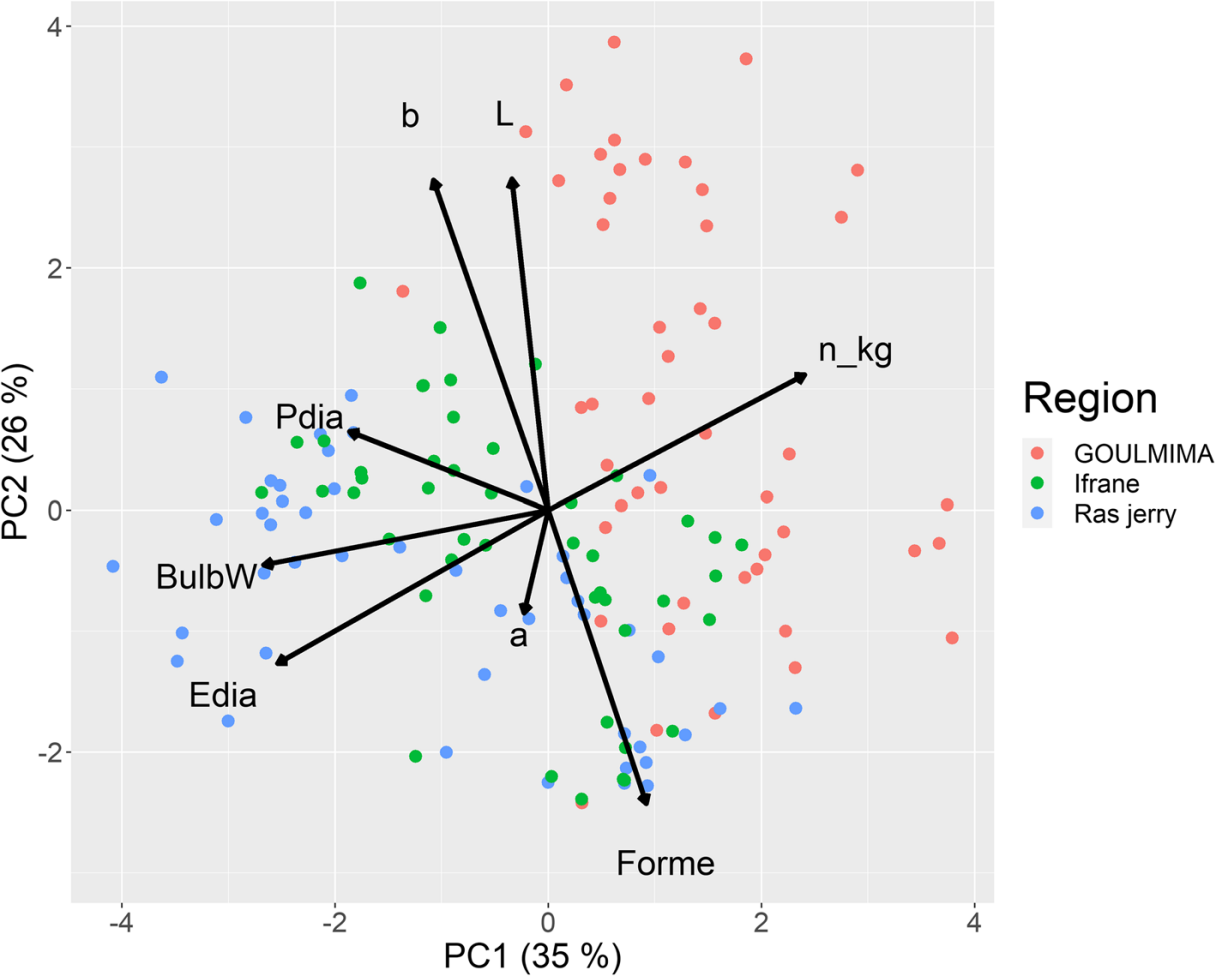
Principal component analysis of phenotypic traits according to regions

**Fig. 7:**
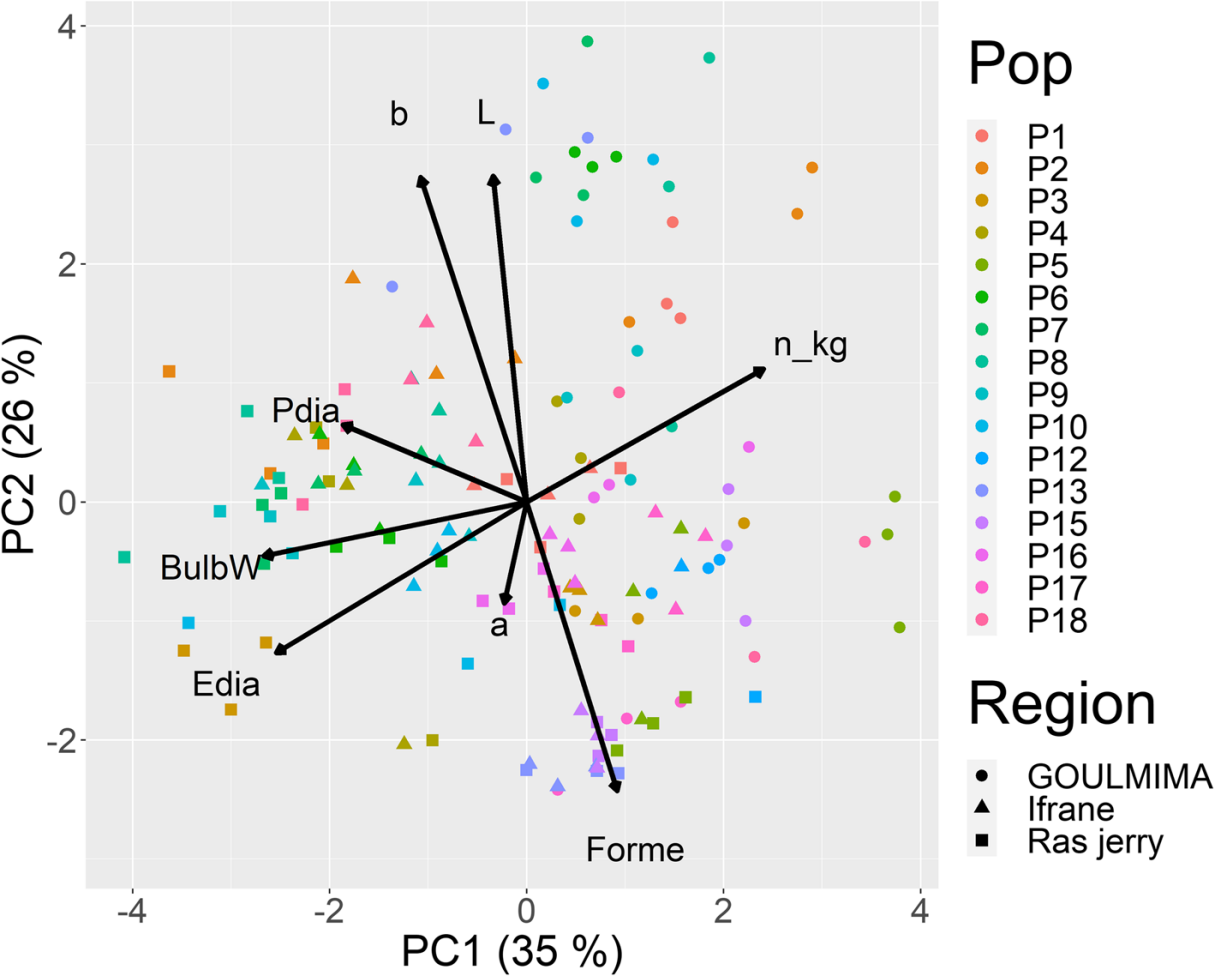
Principal component analysis of phenotypic traits according to ecotypes

A PCA analysis of the phenotypic characteristics revealed that the first component explained 35% of the phenotypic variance and the second component 26%. In addition, traits related to size were strongly associated to the first principal component, whereas the color-related traits (L*, a*, b*) showed a high correlation with the second principal component. In addition, a clear differentiation based on the region where the onions were cultivated can be observed (Fig. 6), e.g., the onions grown in Goulmima were clearly smaller than the onions grown in the other regions. In contrast to the clear grouping between regions, no grouping of the ecotypes (Fig. 7) could be detected, partially due to the genotype × environment interaction.

A Mantel test revealed that there was no significant correlation between the distances based on the ISSR pattern and the distances between the average phenotypes expressed by the ecotypes (*p*-value 0.60).

### Genetic and phenotype diversity correlation

Since both genotypic and phenotypic data were available, the possibility of finding any correlation between a band and a phenotypic trait was tested. The correlation analysis was carried out for the nine different traits and the 127 bands from the 10 ISSR molecular markers. In Table 7, the significant (*p*-value < 0.05) Spearman correlations between the traits and the average marker scores are shown.

**Table 7:** Significant Spearman correlations to analyze the association between molecular markers and the phenotypic traits

	**Pdia**	**Edia**	**Shape**	**BulbW**	***n*** **/kg**	***L*** ^*^	***a*** ^*^	***b*** ^*^
811–6				− 0.58			− 0.56	
811–12					0.53			
811–14				− 0.56				
811–15				− 0.50				
811–17			0.53					
815–1			0.83	− 0.64	0.51			− 0.69
815–2				− 0.53				− 0.58
815–3				− 0.58				
823–2							− 0.67	
823–5			0.55					
823–9	0.53	0.56						
825–3	− 0.50		0.54				− 0.66	
835–3								− 0.64
835–6								− 0.59
835–7								− 0.55
835–8				− 0.56			− 0.54	− 0.59
835–10						− 0.64		− 0.57
840–5	− 0.63		0.54					
840–6			0.67	− 0.55				− 0.65
840–7			0.54					
840–8								− 0.51
840–10		0.53						
842–4			0.52				− 0.67	
842–6	− 0.54		0.56	− 0.55				
842–7			0.60	− 0.55				

Remark that for yield, no significant correlations with the presence of a marker could be found. A positive correlation means that the value of the trait is higher in case a band is present, whereas a negative correlation indicates that the value of the trait increases with a lower average band score.

## Discussion

Variations in genetic and phenotypic diversity are of pivotal importance for breeding. Under these circumstances and for the first time, the genetic and phenotypic diversity in onion cultivated in Morocco were investigated.

Nowadays, molecular markers with a high level of DNA polymorphism contribute to the improvement of vegetable crops around the world [5]. Several molecular markers like ISSR, RAPD, and SSR have been used to study the genetic diversity in the *Allium* genus and also to characterize the genetic relationship between the species [16, 26, 27]. In more recent years, different molecular markers have been introduced to characterize onion populations, like SNP, RAPD, AFPL, ISSR, and SSR [26–31].

Our study addresses for the first time the diversity between onion genotypes from different regions in Morocco. Based on various approaches including genetic distances, AMOVA, LDA, and the genetic diversity between individual plants and between populations, valuable information concerning the available genetic resources was obtained.

The obtained results showed that, based on the ISSR profile, there was a clear subdivision of the 192 onion plants into 16 ecotypes or populations. In addition, the ISSR primers revealed a 100% polymorphism within the onion plants, i.e., each onion plant was characterized by a unique banding pattern. The high level of genetic diversity within onions is confirmed by several researchers using different molecular markers like ISSR, RAPD, and SSR [26, 31] and can be expected especially when outcrossing species like *A. cepa* L. [32]. So, although onion cultivars are highly selected by breeders or farmers, a considerable genetic diversity is still present. However, according to Mukherjee et al. [26], ISSR markers display a higher genetic diversity compared to RAPD markers. The latter markers cover the whole genome, whereas ISSR markers amplify the repetitive regions.

The results of the AMOVA revealed that most of the variation could be attributed to the differences between populations (58%). These results are in contrast with the results of Monteverde et al. [33] who found that most of the marker variation was due to the differences between individuals within the onion populations of Uruguay (66%). Also, for garlic, Kumar et al. [34] concluded, based on SSR markers, that the greater part of the genetic diversity was intra-population, with 84% variation, and only 16% of the variation could be attributed to variation among populations.

Concerning the discriminative power of the various markers, clear differences were observed. The ISSR markers revealed 8 to 18 polymorphic bands for the markers UBC 841 and UBC 840, respectively. The PIC value varied between 0.161 and 0.368. Studying the genetic diversity of selected Indian onion germplasm, the calculated PIC value of ISSR markers ranged between 0.13 and 0.40 [5]. For the SNP markers used to study the genetic diversity in Italian onion landraces, the PIC value varied between 0.00 and 0.38 with an average of 0.29 [29]. In addition, marker UBC 840 turned out to be specific for individuals belonging to population P8. So, UBC 840 can be considered as a population-specific marker.

From the LDA analysis, it was concluded that a new onion plant can be assigned to the correct ecotype with an accuracy of 93% based on its specific ISSR banding pattern.

A comparison of the clustering of the ecotypes/populations according to their genetic makeup and based on the distance between their regions of origin showed some similarities, which could be considered as a genetic breeding base of genotypes belonging to the same origin [5].

Apart from genetic differentiation, plants express phenotypic plasticity, i.e., expressing the optimal phenotype in a certain environment. To detect the phenotypic diversity and a possible association with the genotype, a multi-location field trial was set up. This trial revealed a significant environmental effect (and significant interaction between environment and ecotype) for most of the studied traits. The strong influence of the environment on phenotype is confirmed by Leino et al. [35]. In addition, color-related traits were among the studied traits least influenced by the environment. In contrast to the ISSR marker analysis, a PCA of the phenotypic data showed a grouping of the ecotypes per cultivation environment instead of a grouping by ecotype. The highest positive correlation between a marker score and a trait was obtained for the shape and the UBC 815–1 marker: the higher the average value for this band, the higher the value form. Plus, we noticed a large negative correlation between b* and UBC 815–1, meaning the ecotypes in which this marker is primarily absent have a large value for b*.

## Conclusions

Given the importance of the onion crop in Moroccan agriculture, the present study was carried out to evaluate and characterize, for the first time, the genetic potential of Moroccan onion ecotypes. The analysis of the genetic diversity of 192 Moroccan onion plants, belonging to 16 different ecotypes using ISSR markers profile, reveals a high genetic diversity between onion ecotypes, compared to the genetic variation within onion ecotypes. The clear aggregation of the onion plants into 16 different ecotypes, and the absence of the overlapping in the Neighbor joining tree, confirms that *A. cepa* L. in Morocco consists of well-differentiated ecotypes. Despite the proximity of the geographic origin of some onion ecotypes, a significant genetic diversity was detected.

The study of the genetic diversity of Moroccan onions confirms the efficiency of ISSR markers to gain insight into the population structure. The potential of these molecular markers is represented by high discriminative power between the ecotypes and the high number of polymorphic produced bands. In addition, correct attribution of a new onion plant can be possible using a specific ISSR banding pattern.

Significant phenotypical diversity was revealed through the characterization of the onion ecotype phenotype under three different environments in Morocco. Furthermore, the obtained results affirm a significant influence of the environment on the majority of phenotypical traits, and the genotype influence of all studied characters, especially the coloration components. Next to that, a strong interaction effect between environment and genotype was also confirmed. In addition, the present study showed that onion ecotypes are different essentially by the size traits, followed by the coloration characters. Finally, some significant associations between marker scores and phenotypic traits were detected. This combination of genetic and phenotypic data on onions is important to select breeding populations needed for future breeding programs.

## Data Availability

The datasets used and/or analyzed during the current study are available from the corresponding author on reasonable request.

## Abbreviations

ISSR: Inter-simple sequence repeat
PIC: Polymorphic information content
*A. cepa* L.: *Allium cepa* L.
UBC: University of British Columbia
PCA: Principal component analysis
LDA: Linear discriminant analysis
Pdia: Polar diameter
Edia: Equatorial diameter
*H*^2^: Heritability
Bp: Base pair

## References

[1] Food and Agriculture Organization of the United Nations (2020) FAOSTAT Database. http://fao.org/faostat/en/#data/QC. Accessed 23 May 2022

[2] Griffiths G, Trueman L, Crowther T, Thomas B, Smith B (2002). Onions: a global benefit to health. Phytother Res.

[3] McCallum J, Havey MJ, Shigyo M, McManus MT (2008). Molecular approaches to characterizing and improving bulb composition in onion. Acta Hortic.

[4] Lee Y-R, Kim CW, Han J, Choi HJ, Han K, Lee ES, Kim D-S, Lee J, Siddique MI, Lee H-E (2021). Genotyping-by-sequencing derived genetic linkage map and quantitative trait loci for sugar content in onion (Allium cepa L). Plants..

[5] Sudha GS, Ramesh P, Sekhar AC, Krishna TS, Bramhachari PV, Riazunnisa K (2019). Genetic diversity analysis of selected onion (Allium cepa L.) germplasm using specific RAPD and ISSR polymorphism markers. Biocatal Agric Biotechnol.

[6] Walters SA, Bouharroud R, Mimouni A, Wifaya A (2018). The deterioration of Morocco’s vegetable crop genetic diversity: an analysis of the Souss-Massa region. Agriculture.

[7] Enciso J, Jifon J, Anciso J and Ribera L (2015) Productivity of onions using subsurface drip irrigation versus furrow irrigation systems with an internet based irrigation scheduling program. Int J Agron 6. 10.1155/2015/178180

[8] Karic L, Golzardi M, Glamoclija P, Sutkovic J (2018) Genetic diversity assessment of Allium cepa L. cultivars from Bosnia and Herzegovina using SSR makers. Genet Mol Res 17(1): gmr16039870. 10.4238/gmr16039870

[9] Mitrová K, Svoboda P, Ovesná J (2015). The selection and validation of a marker set for the differentiation of onion cultivars from the Czech Republic. Czech Journal of Genet Plant Breed.

[10] Khar A, Lawande KE, Negi KS (2010). Microsatellite marker based analysis of genetic diversity in short day tropical Indian onion and cross amplification in related Allium spp. Genetics Resour Crop Evol.

[11] Chinnappareddy LRD, Khandagale K, Chennareddy A, Ramappa VG (2013). Molecular markers in the improvement of Allium crops. Czech J Genet Plant Breed.

[12] De Riek J, Calsyn E, Everaert I (2001). AFLP based alternatives for the assessment of distinctness, uniformity and stability of sugar beet varieties. Theor Appl Genet.

[13] Terzopoulos PJ, Bebeli PJ (2008). Genetic diversity analysis of Mediterranean faba bean (Vicia faba L.) with ISSR markers. Field Crops Research.

[14] Vijayan K (2005). Inter simple sequence repeat (ISSR) polymorphism and its application in mulberry genome analysis. Int J Ind Entomol.

[15] Reddy PM, Sarla N, Siddiq EA (2002). Inter simple sequence repeat (ISSR) polymorphism and its application in plant breeding. Euphytica.

[16] Son JH, Park KC, Lee S, Kim JH, Kim NS (2012). Species relationships among Allium species by ISSR analysis. Hortic Environ Biotechnol.

[17] Salimath SS, de Oliveira AE, Godwin JD, Bennetzen JL (1995). Assessment of genome origins and genetic diversity in the genus Eleusine with DNA markers. Genomics.

[18] Joshi SP, Gupta YS, Aggarwal RK, Ranjekar PK, Brar DS (2000). Genetic diversity and phylogenetic relationship as revealed by inter-simple sequence repeat (ISSR) polymorphism in the genus Oryza. Theor Appl Genet.

[19] Houmanat K, Charafi J, Mazouz H, El Fechtali M, Nabloussi A (2016). Genetic diversity analysis of safflower (Carthamus tinctorius L) accessions from different geographic origins using ISSR markers. Int J Agri Biol.

[20] Levin I, Gilboa N, Yeselson E, Shen S, Schaffer AA (2000). Fxr, a major locus that modulates the fructose to glucose ratio in mature tomato fruits. Theor Appl Genet.

[21] McSweeney C, New M, Lizcano G (2010) UNDP Climate Change Country Profiles: Morocco. Available: https://www.geog.ox.ac.uk/research/climate/projects/undpcp/index.html?country=Morocco&d1=Reports

[22] ISTA (2015). International rules for seed testing.

[23] R Core Team (2018) R: a language and environment for statistical computing. R Foundation for Statistical Computing, Vienna, Austria. https://www.R-project.org/

[24] Roldán-Ruiz I, Dendauw J, Van Bockstaele E, Depicker A, De Loose M (2000). AFLP markers reveal high polymorphic rates in ryegrasses (Lolium spp.). Mol Breeding.

[25] Jaccard P (1908). Nouvelles recherches sur la distribution florale. Bulletin de la Société Vaudoise des Sciences Naturelles.

[26] Mukherjee A, Sikdar B, Ghosh B, Banerjee A, Ghosh E, Bhattacharya M, Roy CS (2013). RAPD and ISSR analysis of some economically important species, varieties, and cultivars of the genus Allium (Alliaceae). Turk J Bot.

[27] Chen S, Chen W, Shen X, Yang Y, Qi F, Liu Y, Meng H (2014). Analysis of the genetic diversity of garlic (Allium sativum L.) by simple sequence repeats and inters simple sequence repeat analysis and agro-morphological traits. Biochem Syst Ecol.

[28] Lee JH, Arif R, Sathishkumar N, Hee-Jeong J, Ill-Sup N (2018). Varietal identification of open-pollinated onion cultivars using a nanofluidic array of single nucleotide polymorphism (SNP) markers. Agronomy.

[29] Villano C, Esposito S, Carucci F, Iorizzo M (2018). High-throughput genotyping in onion reveals structure of genetic diversity and informative SNPs useful for molecular breeding. Mol Breeding.

[30] Santos CAF, Oliveira VR, Rodrigues MA, Ribeiro HLC, Silva GO (2011). Genetic similarity among onion cultivars of different types and origins, based on AFLP markers. Hortic Bras.

[31] Mallor C, Arnedo-Andrés MS, Garcés-Claver A (2014). Assessing the genetic diversity of Spanish Allium cepa landraces for onion breeding using microsatellite markers. Sci Hortic.

[32] Taylor A, Teakle GR, Walley PG (2019). Assembly and characterisation of a unique onion diversity set identifies resistance to Fusarium basal rot and improved seedling vigour. Theor Appl Genet.

[33] Monteverde E, Galván GA, Speranza P (2014). Genetic diversification of local onion populations under different production systems in Uruguay. Plant Genet Resour.

[34] Kumar M, Sharma V, Kumar V, Sirohi U, Chaudhary V, Sharma S, Gautam S, Naresh RK, Yadav HK, Sharma S (2019). Genetic diversity and population structure analysis of Indian garlic (Allium sativum L.) collection using SSR markers. Physiol Mol Biol Plants.

[35] Leino MW, Solberg SØ, Tunset HM, Fogelholm J, Strese EK, Hagenblad J (2018). Patterns of exchange of multiplying onion (Allium cepa L. Aggregatum-Group) in Fennoscandian home gardens. Economic Botany..

